# Open questions: Zombie projects, translational research, and the real secret of the inside of the cell

**DOI:** 10.1186/1741-7007-11-97

**Published:** 2013-09-02

**Authors:** Gregory A Petsko

**Affiliations:** 1 Weill Cornell Medical College, New York, NY 10065, USA

## 

It may be a sign of incipient old age, but I find myself increasingly convinced that the key question in biology these days is philosophical, not scientific. It is the question of what balance should exist, given that the financial pie is not only finite but likely to be shrinking for some time, between large-scale, top-down, ‘big science’ projects that are primarily aimed at information gathering, and hypothesis-driven, individual-investigator initiated ‘little science’ projects. The seesaw has tilted pretty heavily in favor of the big stuff lately. I have nothing against such programs in principle, but it seems to me that in practice there are too many of dubious merit and low benefit-to-cost ratio. They are never pitted against small science projects when they are reviewed, and because they are attractive to administrators - they produce lots of data that can be shown proudly to superiors, politicians and citizens - they become very difficult to kill, even if the data are not very useful or the original mission has been fulfilled, because too many influential reputations and comfortable livelihoods are vested in their continuation. As I see it, there is a danger that these ‘zombie programs’ will take over biology, and investigator-initiated research, which is where most breakthroughs come from, is already getting short shrift as a consequence. It’s time we stop letting that happen by default, and ask how priorities in biomedical research should be set, and by whom.

Two other, interrelated philosophical questions are: (1) whether biological research should focus predominantly on mammalian cells and organisms - the direction it seems to be heading - or whether there is, as I firmly believe, still a large place for work in model organisms; and (2) whether the distinction between ‘basic’ and ‘translational’ research is, as I have asserted elsewhere [1], an artificial and unproductive one, and therefore should be eliminated. Both these questions are reflections of the tension between the immediately practical and the more open-ended. As long as we believe that ‘translational’ research has to be given special emphasis, we will tend to undervalue work that lacks an obvious, immediate payoff, and to favor studies in human cells that address human diseases. It may be that this is what we should do, but if so, we should make a deliberate decision to do it after carefully considering the questions as a community, not simply allow it to happen by fiat because of our apathy or fatalism.

Lest you think this is all too metaphysical, I do have a key scientific question to offer. I think one of the most important unsolved problems in biology is what the interior of the cell really looks like. This holds for both prokaryotic and eukaryotic cells. We’ve all seen those beautiful cartoons of the crowded environment inside a bacterium, and have watched mesmerizing videos like The Inner Life of the Cell [2], but those are artists’ conceptions - based on sound science, to be sure, but still rife with conjecture. My own prejudice is, to borrow a phrase, that the inside of any cell is not only more organized than we imagine, but more organized than we can possibly imagine (at present, anyway). But the point is that neither I nor anybody else knows. Answering this question is vital for the future of biochemistry as well as biology, because a century of studies on the function of, for example, enzymes has been carried out in dilute aqueous solution, where the substrates were vastly in excess of the protein. The situation inside a cell is almost certainly the opposite, a difference that has profound implications for our understanding of both rates and regulation. The interior of a cell probably resembles Times Square on New Year’s Eve, not the Australian outback. But how chaotic are things in that jam-packed square? Are the protein components of, say, metabolic pathways randomly dispersed, as textbooks seem to assume, or are there dynamic complexes in which many of the participants in a metabolic pathway come together in space (as has been shown to occur in signaling pathways)? Do any large molecules passively diffuse inside a cell, or is everything transported along cytoskeletal railroad tracks to specific locations - and if so, what determines those locations? These and related questions are a subset of the big one, maybe the biggest one facing biology in the 21st century: what is it really like in there?
